# A Review of Extraction, Structural Properties, Biological Activities of *Dendrobium chrysotoxum* Polysaccharides

**DOI:** 10.1002/fsn3.71951

**Published:** 2026-05-29

**Authors:** Xingshuai Zhang, Zhaoyuan Xu, Jialin Chen, Weitong Zhang, Lihang Xie

**Affiliations:** ^1^ State Key Laboratory of Metabolic Dysregulation & Prevention and Treatment of Esophageal Cancer, Tianjian Laboratory of Advanced Biomedical Sciences, School of Convergence Medicine Zhengzhou University Zhengzhou China; ^2^ School of Life Sciences Zhengzhou University Zhengzhou China; ^3^ The First Clinical Medical College Zhengzhou University Zhengzhou China; ^4^ The First Affiliated Hospital Sun Yat‐sen University Guangzhou China; ^5^ School of Life Sciences Central South University Changsha China; ^6^ Henan Funiu Mountain Biological and Ecological Environment Observatory Zhengzhou University Zhengzhou China

**Keywords:** bioactivities, *Dendrobium chrysotoxum*, extraction and purification, polysaccharides, structural characterization

## Abstract

*Dendrobium chrysotoxum* Lindl., an important medicinal plant, contains polysaccharides (DCP) as its principal bioactive components. This review systematically outlines recent advances in the extraction, purification, structural characteristics, biological activities, and future prospects of DCP. For extraction, conventional hot water extraction followed by ethanol precipitation remains common but is limited by high energy consumption and prolonged duration. Advanced techniques, such as enzyme‐assisted extraction (yielding 8.41% under optimized conditions) and a combined ultrasonic‐hot water method (achieving 20.41% purity), significantly enhance efficiency. Purification generally involves deproteinization, removal of low‐molecular‐weight impurities, and fractionation via column chromatography to obtain homogeneous polysaccharide fractions. Structurally, DCP are heteropolysaccharides primarily consisting of glucose and mannose in varying molar ratios, with molecular weights ranging from 10^4^ to 10^6^ Da. Their backbone is mainly composed of (1→4)‐linked Manp and Glcp residues, featuring branching and partial acetylation. Biologically, DCP exhibit a broad spectrum of activities, including antioxidant and anti‐aging effects, immunomodulation, and organ protection. Additionally, DCP show hypoglycemic and antitumor potential, inhibiting proliferation of SPC‐A‐1 and HeLa cells through apoptosis induction. Despite these findings, challenges remain including inefficient extraction and insufficient understanding of the structure–activity relationships and molecular mechanisms underlying DCP's diverse biological activities. Future research should prioritize innovative extraction technologies, optimized purification, in‐depth mechanistic investigations, and the development of practical applications.

## Graphical abstract

Overview of extraction, biological activities, and application prospects of DCP. (a) Collection, weighing, shearing, and grinding of *D. chrysotoxum* stems. (b) Aqueous extraction, ultracentrifugation, drying, and filtration to obtain purified DCP. (c) Mechanistic studies and preclinical or retrospective clinical investigations of DCP bioactivities. (d) Structural characteristics and main biological functions of DCP, including hypoglycemic, immunomodulatory, organ‐protective, anti‐aging, and anti‐tumor effects. (e) Prospective applications based on new extraction technologies and clinical potentials in diabetes, cardiovascular diseases, tumors, organ injury, inflammation, and neurodegenerative disorders.

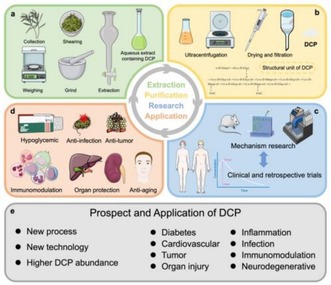

## 1 Introduction

*Dendrobium chrysotoxum* Lindl. (*Orchidaceae*), a perennial epiphytic herb, is a valued orchid with a history of use dating back to *Shennong's Classic of Materia Medica* (Zhou et al. 2018). The genus *Dendrobium* encompasses rich botanical diversity, with approximately 78 documented species to date. *D. chrysotoxum* is native to southern and western Yunnan, China, and is also distributed across India, Myanmar, Thailand, Laos, and Vietnam. Recognized as an official medicinal material in the *Pharmacopeia of the People's Republic of China* (2020 Edition), it is traditionally used to enhance immune function, stimulate saliva secretion, suppress pharyngitis, alleviate heat syndrome, treat chronic superficial gastritis, and replenish yin (China 2020; Yue et al. 2020).

The main reason why *D. chrysotoxum* exhibits a wide range of biological effects is mainly that it contains various bioactive substances such as polysaccharides. *D. chrysotoxum* is rich in diverse bioactive components in its stems and flowers, with the stems containing abundant water‐soluble polysaccharides and the flowers possessing a variety of functional substances including flavonoids, alkaloids, polyphenols, carotenoids and free amino acids. As the core bioactive component of *D. chrysotoxum*, polysaccharides have a high proportion in the stem, where the dominant polysaccharide fraction DCPP‐I‐a accounts for 79.5% of the main stem polysaccharide component, and the flower also has a considerable polysaccharide content (Sun et al. 2013; Xu et al. 2025). Polysaccharides, composed of monosaccharide units linked by glycosidic bonds, are essential macromolecules ubiquitous in animals, plants, and microorganisms (Chen and Huang 2018b; Li et al. 2018). In *D. chrysotoxum*, polysaccharides primarily function as structural components of the cell wall and are most abundant in the stems (Chen et al. 2001). As the principal bioactive constituent of *D. chrysotoxum*, these polysaccharides exhibit a wide spectrum of pharmacological activities, including antioxidant (Chen et al. 2022), anti‐aging (Li 2016), hypoglycemic (Zhao et al. 2007), immunomodulatory (Pan et al. 2015), antitumor (Sun et al. 2013), hepatoprotective (Qian et al. 2015), renoprotective (Hao et al. 2023), and gastrointestinal protective (Wang et al. 2024) effects. Over recent decades, ongoing research into the biological functions of *D. chrysotoxum* polysaccharides (DCP) has drawn increasing scientific interest.

Despite the growing body of evidence, a comprehensive narrative review summarizing the extraction, purification, structural features, and bioactivities of DCP remains lacking. This article aims to comprehensively consolidate current knowledge in these areas and propose future research directions, thereby providing a foundational reference for further exploration and utilization of these polysaccharides.

## 2 Extraction and Purification of *D. chrysotoxum* Polysaccharides

**FIGURE 1:**
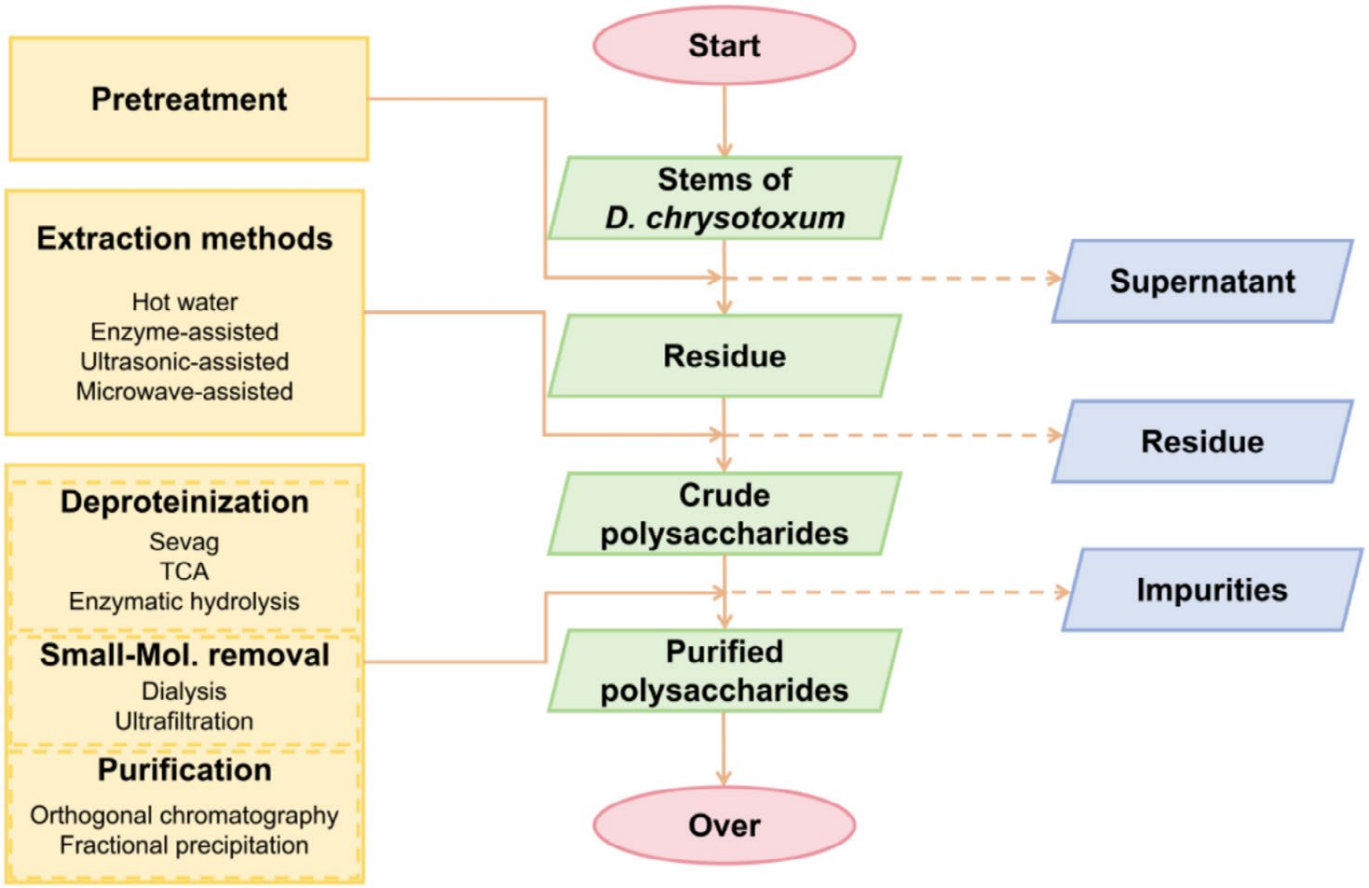
The general extraction and purification procedures of DCP.

To obtain polysaccharides with enhanced bioactivity from *D. chrysotoxum* and to facilitate further investigation into their structural characteristics and pharmacological functions, it is essential to disrupt the cellular architecture of the plant's stems. Following the extraction of crude polysaccharides, subsequent separation and purification steps are necessary to eliminate impurities such as pigments, proteins, and low‐molecular‐weight compounds. Ultimately, homogeneous polysaccharide fractions can be isolated from the polysaccharide mixture using column chromatography, providing purified materials for downstream scientific studies (Figure 1).

### 2.1 Extraction Methods for DCP

To date, several extraction techniques for DCP have been documented, primarily including hot water extraction followed by ethanol precipitation, ultrasonic‐assisted extraction, enzyme‐assisted extraction, and microwave‐assisted extraction. This section summarizes the principles, operational procedures, advantages, and limitations associated with these methods (Table 1).

**TABLE 1:** Extraction methods for DCP.

Extraction methods	The raw material organs	Pretreatment	Temperature	Time	Liquid‐to‐solid ratio	Power	Enzyme	Extraction efficiency (%) or polysaccharide content (g/g)	References
Hot water extraction and ethanol precipitation	Flowers	NA	80°C	2 h	100:1	NA	NA	0.25 g/g	Fan et al. (2022); Xu et al. (2025)
	Stems	NA	100°C	1 h	16:1	NA	NA	3.60%	Meng et al. (2013)
		NA	80°C	1.5 h	50:1	NA	NA	5.28%	Wang (2013)
		Acetone	80°C	4 h	20:1	NA	NA	6.73%	Pan et al. (2015)
Enzyme‐assisted extraction		Acetone	40°C	3 h	25:1	NA	Cellulase (10 g/L)	8.41%	Pan et al. (2015)
Ultrasonic‐assisted extraction		Petroleum	40°C ultrasonic, 80°C reflux	45 min ultrasonic, 1 h reflux	12.5:1	135 W	NA	0.20 g/g	Li (2008)
Microwave‐assisted extraction		NA	80 ~ 100°C	1 ~ 3 h	4:1 ~ 8:1	650 ~ 750 W	NA	18.3% ~ 19.1%	Huang, Peng, et al. (2018)

*Note:* NA: There are currently no literature reports available.

#### 2.1.1 Hot Water Extraction and Ethanol Precipitation

Hot water extraction and ethanol precipitation is a conventional and widely applied method for extracting polysaccharides from *D. chrysotoxum*. This technique employs water as the extraction solvent, followed by impurity removal via ethanol‐induced precipitation. The resulting crude polysaccharides are collected through drying or freeze‐precipitation. A typical procedure involves grinding the stems of *D. chrysotoxum*, followed by repeated hot water extraction at 80°C to 100°C (Hao et al. 2023; Meng et al. 2013; Pan et al. 2015; Shang et al. 2021; Wang 2013). The extract is then filtered, centrifuged, and concentrated (Pan et al. 2015). Subsequently, 80% ethanol is added, and the mixture is stored overnight at 4°C to precipitate polysaccharides (Li 2008). After the second centrifugation, the precipitate was washed alternately with anhydrous ethanol and acetone to obtain the crude polysaccharides (Yue et al. 2020).

Various researchers have adopted this method under differing conditions. For instance, Xu et al. conducted hot water extraction of polysaccharides from dried *D. chrysotoxum* flowers with a liquid‐to‐solid ratio of 100:1 (distilled water is used for all extractions in this section) at 80°C, for 2 h, and determined the polysaccharide content in the floral samples to be 0.25 g/g on a dry weight basis by the phenol‐sulfuric acid colorimetric method, using glucose as the standard reference substance to establish a calibration curve and calculate the polysaccharide content in terms of glucose equivalent (Xu et al. 2025). However, most studies have focused on the stems as the raw material. With a liquid‐to‐solid ratio of 16:1 and extraction at 100°C, for 1 h, a polysaccharide yield of 3.6% was achieved (Meng et al. 2013). Under more optimized conditions—liquid‐to‐solid ratio of 50:1, temperature of 80°C, and duration of 1.5 h—the extraction efficiency increased to 5.28% (Wang 2013). Pretreatment of the raw material, such as defatting and decolorization, can further improve extraction yields. For example, Pan et al. treated dried stem powder with acetone for 24 h to remove pigments and lipids prior to extraction. Under conditions of a 20:1 liquid‐to‐solid ratio, 80°C, and 4 h, a yield of 6.73% was obtained (Pan et al. 2015). Similar pretreatments using petroleum ether (concentration unspecified in the study) or 80% ethanol have also been reported to enhance the hot water extraction efficiency of *D. chrysotoxum* polysaccharides, with the respective extraction efficiency reaching 4.26% and 3.72% (Hao et al. 2023; Shang et al. 2021). These findings indicate that factors such as liquid‐to‐solid ratio, extraction time, temperature, and raw material pretreatment significantly influence the performance of this method.

Although this approach is straightforward and suitable for large‐scale production, it generally requires high temperatures and large volumes of solvent, making it time‐ and labor‐intensive (He et al. 2022). Elevated temperature may induce the conformational relaxation of polysaccharides, thereby reducing their binding affinity to target proteins and consequently diminishing their biological activity (Chen et al. 2024). Prolonged extraction can co‐extract excessive impurities, complicating subsequent purification (Ke 2015). Therefore, combining this method with other extraction technologies is often necessary to broaden its applicability and address these limitations.

#### 2.1.2 Enzyme‐Assisted Extraction

Compared with traditional hot water extraction, enzyme‐assisted extraction is more suitable for the extraction of DCP, as it effectively overcomes the shortcomings of low yield, long extraction time and structural degradation of polysaccharides caused by high temperature. Cellulase can degrade the cellulose in the cell wall of *D. chrysotoxum* to form porous structures, which facilitates solvent penetration and promotes the release of intracellular DCP, while functioning under mild conditions to avoid thermal degradation and preserve the structure and bioactivity of DCP; pectinase can decompose the intercellular adhesive pectin, loosen cell connections and eliminate the diffusion barrier for DCP release. Amylase and ligninase are not used due to the low substrate content in *D. chrysotoxum*, since they cannot improve extraction efficiency but increase the process cost and subsequent purification difficulty (Baez and Bacete 2023; Fan et al. 2022).

Commonly used enzymes include cellulase and pectinase. Pan et al. optimized extraction parameters using single‐factor and orthogonal L_9_ (3^4^) experimental designs. Under optimal conditions—pH 5.5, temperature of 40°C, cellulase concentration of 10 g/L, extraction time of 3.0 h, and Liquid‐to‐Solid Ratio of 25:1—the polysaccharide yield reached 8.41% (Pan et al. 2015). For the purified DCP fractions separated by column chromatography after enzyme‐assisted extraction, gel permeation chromatography (GPC) can be used for absolute quantitative analysis after molecular weight calibration with dextran standards, and the homogeneity of polysaccharide fractions can be evaluated simultaneously (Shang et al. 2021; Sun et al. 2013). Similarly, polysaccharides from other species of the genus *Dendrobium* can be extracted via enzyme‐assisted methods. Fan Yijun and colleagues reported that the extraction efficiency of polysaccharides from *Dendrobium devonianum* using cellulase was significantly higher than that using pectinase. The optimal enzymolysis conditions were determined as follows: enzyme concentration 0.94%, enzymolysis temperature 51.06°C, and pH 4.95, under which the polysaccharide extraction yield reached 15.76% (Fan et al. 2022). Lian He et al. adopted cellulase‐assisted extraction for polysaccharides from *Dendrobium officinale*, and the pH was adjusted to 5.0 ± 0.2, followed by extraction with 0.15% (m/v) cellulase (10,000 U/g) at 55°C, for 3 h, affording an extraction yield as high as 18.50% (Fan et al. 2022).

Enzyme‐assisted extraction is performed under mild conditions, effectively disrupting cell walls and hydrolyzing proteins, which promotes polysaccharide release and enhances both yield and bioactivity (Song et al. 2018). However, the method is associated with high production costs and stringent requirements for reaction conditions.

#### 2.1.3 Ultrasonic‐Assisted Extraction

Ultrasonic‐assisted extraction utilizes cavitation, thermal, and mechanical effects generated by ultrasound to disrupt cellular structures, thereby facilitating the release of bioactive compounds and improving mass transfer (Mena‐García et al. 2019; Wen et al. 2018). Studies indicate that ultrasonic extraction alone (40°C for 45 min, liquid‐to‐solid ratio of 40:1, v/w) yields a polysaccharide content of 0.1046 g/g for *D. chrysotoxum*, which is lower than that obtained by the conventional hot water reflux extraction method (80°C for 3 h per extraction, repeated twice with a total extraction time of 6 h, 0.19 g/g) (Li 2008). It should be noted that the temperature and time parameters of the single hot water extraction are quite different from those of the combined extraction: the single hot water extraction adopts a prolonged 6 h reflux at a constant 80°C, while the combined method shortens the hot water reflux period to 1 h after ultrasonic pretreatment. Notably, the combination of ultrasonic pretreatment and subsequent hot water reflux extraction significantly increases the polysaccharide content to 0.20 g/g, with the optimized extraction conditions as follows: a liquid‐to‐solid ratio of 40:1 (v/w), ultrasonic treatment at 40°C for 45 min, followed by hot water reflux extraction at 80°C for 1 h under pH 5 (Li 2008). Further orthogonal test analysis revealed that among the key extraction factors, extraction temperature exerts the most significant influence on the polysaccharide yield, followed by the liquid‐to‐solid ratio, extraction time, and pH value (Li 2008).

An alternative hot water‐ultrasonication combined extraction strategy, which is superior to the conventional single hot water extraction in improving polysaccharide extraction efficiency by full release of intracellular water‐soluble polysaccharides, was employed for the extraction of crude polysaccharides from *D. chrysotoxum* stems (Sun et al. 2013). Specifically, the pretreated plant residue was first subjected to reflux extraction with double‐distilled water at 80°C for 1 h per time for two consecutive times to initially dissolve the free water‐soluble polysaccharides; the residual material was then further subjected to ultrasonic‐assisted extraction with double‐distilled water at 40°C for another two times (1 h per time), where the ultrasonic cavitation and mechanical effects disrupt the plant cell wall structure to release more bound polysaccharides. All the aqueous extracts obtained from the two extraction steps were combined and subsequently subjected to ethanol precipitation for the recovery of crude polysaccharides (Sun et al. 2013).

Ultrasonic‐assisted extraction offers several advantages, including environmental friendliness, reduced organic solvent usage, energy efficiency, shortened extraction time, high yield, broad applicability, good reproducibility, and minimal thermal degradation of heat‐sensitive components (Chemat et al. 2017; Tiwari 2015). However, temperature control during ultrasonication remains challenging, which may hinder industrial scalability. Moreover, prolonged exposure to ultrasound may cause structural degradation of polysaccharides, adversely affecting their bioactivity (Ren et al. 2019). This degradation primarily results from mechanical bond breakage and cavitation effects, where the collapse of cavitation bubbles generates strong shear forces and localized high pressure that cleave glycosidic bonds and disrupt hydrogen‐bonding networks (Dou et al. 2023; Wang, Zhou, et al. 2023). Additionally, ultrasound‐induced free radicals may further promote chain scission and alter polysaccharide conformation.

#### 2.1.4 Microwave‐Assisted Extraction

Microwave‐assisted extraction is an emerging technology for polysaccharide extraction, characterized by high selectivity, rapid processing, low solvent and energy consumption, and short extraction times. Huang et al. introduced a microwave puffing technique for cell wall disruption as a pretreatment step: fresh *D. chrysotoxum* stems are cleaned, air‐dried, and further dried at 85°C–95°C for 3.5–4.0 h to reduce moisture content to 15%–30% (optimally 20%–25%). The stems are cut into 3.5–4.0 cm segments and subjected to microwave puffing at 650–750 W for 35–45 s. The resulting material is then ground and sieved through 100–300 mesh. For extraction, water amounting to 4–8 times the sample weight is used, and percolation or reflux extraction is performed 3–4 times at 80°C–100°C for 1–3 h per cycle. The extracts are combined, filtered, and concentrated to a relative density of 1.1–1.3, then dried and pulverized to obtain the final product. This microwave puffing pretreatment creates a porous structure, significantly enhancing polysaccharide leaching efficiency, with yields reaching 18.3%–19.1% of the sample weight (Huang, Peng, et al. 2018).

Although highly efficient, the method requires specialized equipment, and extended exposure may cause localized overheating, leading to polysaccharide degradation and structural alteration.

### 2.2 Purification of DCP

The separation and purification of DCP are essential prerequisites for in‐depth structural characterization and pharmacological evaluation. Crude polysaccharides obtained by standard extraction procedures typically contain various impurities, including fat‐soluble components, proteins, pigments, and low‐molecular‐weight substances. These contaminants can interfere with biological activity, compromise stability, and affect the overall quality of the polysaccharides. Purification is therefore necessary to obtain homogeneous polysaccharide fractions and to elucidate their structural features. This section reviews the primary techniques for deproteinization, removal of small‐molecule impurities, and final purification of crude DCP (Table 2).

**TABLE 2:** Purification methods for DCP.

Name	Deproteinization	Small‐mol. removal	Purification	References
DCP‐W4	Sevag method	Dialysis	Stepwise ethanol precipitation	Shang et al. (2021)
			DEAE cellulose, Sephadex G‐100	
DCPP	Pepsin‐Sevage method	Dialysis	NA	Li (2008)
DCP‐1	Trypsin‐Sevage method	Dialysis	DEAE cellulose‐52, Sephadex G‐100	Hao et al. (2023)
DCP‐E, DCP‐H	Sevag method	Dialysis	NA	Pan et al. (2015)
DCP‐W1	Papain‐Sevag method	Dialysis	DEAE cellulose, Sephadex G‐100	Wang (2013)
DCP‐W2, DCP‐W3	Papain‐Sevag method	Dialysis	DEAE cellulose, Sephacryl S‐400	

*Note:* NA: There are currently no literature reports available. Name: The names of polysaccharide fractions listed in the table are the original designations used in the respective references.

#### 2.2.1 Deproteinization

Proteins in crude DCP extracts may adversely influence polysaccharide quality and hinder structural and bioactivity analyses. Commonly employed deproteinization strategies include the Sevag method (Guo et al. 2023), trichloroacetic acid (TCA) precipitation (Chen and Huang 2018a), and enzymatic hydrolysis (Shi et al. 2023).

The Sevag method, a conventional technique, utilizes a chloroform–n‐butanol mixture to denature proteins, forming insoluble complexes that can be separated (Pan et al. 2015). While cost‐effective, this approach is labor‐intensive and often requires multiple treatment cycles (Zhang et al. 2016). The TCA method induces protein denaturation and the formation of insoluble TCA‐protein salts; however, high TCA concentrations risk hydrolyzing glycosidic bonds, potentially degrading polysaccharide structure and reducing yield (Wang 2013; Xiao et al. 2018).

Enzymatic hydrolysis eliminates proteins under mild conditions using specific proteases, offering high selectivity and minimal damage to polysaccharides. Its main limitation lies in the relatively high cost (Yan et al. 2016). To improve efficiency and preserve polysaccharide integrity, combined methods are often adopted. For instance, Li used 0.4 mL of 10 mg/mL pepsin solution for enzymatic treatment, while trypsin or papain have also been employed to hydrolyze both free and partially bound proteins, followed by Sevag reagent to remove residual enzymatic and free proteins (Hao et al. 2023; Li 2008). Furthermore, for the deproteinization of water‐soluble polysaccharides from *D. chrysotorum*, four methods were compared by Wang (2013) with protein removal rate and polysaccharide loss rate as evaluation indexes. The Sevag method achieved a 62.93% protein removal rate with a 27.72% polysaccharide loss rate; the TCA method had a 65.14% protein removal rate but the highest polysaccharide loss rate (34.36%); the papain method showed the lowest polysaccharide loss rate (6.66%) yet the poorest protein removal efficiency (60.71%). Notably, the papain‐Sevag combined method reached the highest protein removal rate (70.68%) and the minimal polysaccharide loss rate (6.28%), making it the optimal method for its balanced high efficiency and low polysaccharide loss (Wang 2013).

Recent investigations have explored novel deproteinization strategies for Dendrobium polysaccharides, such as freeze–thaw treatment, which denatures and aggregates proteins through repeated freezing and thawing for subsequent removal by centrifugation; dialdehyde cellulose adsorption, in which aldehyde groups covalently bind to the amino groups of proteins; and magnetic chitosan microsphere‐based methods, where positively charged chitosan adsorbs negatively charged proteins and allows easy separation with a magnetic field, aiming to enhance efficiency and environmental sustainability (He et al. 2019; Tang et al. 2020).

#### 2.2.2 Removal of Small‐Molecule Impurities

Following deproteinization, crude polysaccharide preparations still contain low‐molecular‐weight impurities such as monosaccharides, oligosaccharides, and inorganic salts. These are commonly removed by dialysis or ultrafiltration. Ultrafiltration is widely applied in macromolecular separations due to its high retention rate, cost‐effectiveness, operational safety, and environmental friendliness. However, its application in purifying DCP remains limited, with dialysis being more frequently reported.

For example, Li Ting et al. applied a double‐dialysis process: the deproteinized polysaccharide solution was dialyzed (MWCO 2 kDa) first against running water for 48 h to eliminate inorganic salts and small oligosaccharides, then against distilled water for 24 h for further purification (Li 2008). The retentate was subsequently concentrated under reduced pressure. Although simple, dialysis is time‐consuming and low‐throughput. Moreover, linear polysaccharide molecules may partially penetrate the membrane, leading to sample loss (Wang, Fan, et al. 2023).

#### 2.2.3 Purification

Even after impurity removal, DCP remains a mixture of components varying in molecular weight and structure. Further separation into individual fractions is critical for precise structural analysis (He et al. 2022; Shang et al. 2021).

Column chromatography is the most widely used purification technique, primarily involving size‐exclusion (gel filtration) and ion‐exchange chromatography. Acidic or neutral polysaccharides are often first separated by polarity using ion‐exchange columns (e.g., DEAE‐Cellulose), followed by fractionation by molecular size using gel filtration media (e.g., Sephadex G‐series, Sephacryl S‐series) (Wang 2013). Moreover, Sephadex G100, a dextran gel, was used to purify the low‐molecular‐weight fraction DCP‐W1, while Sephacryl S400, a copolymer gel with good mechanical stability and a wide separation range, was applied for the high‐molecular‐weight fractions DCP‐W2 and DCP‐W3 to obtain homogeneous polysaccharides (Wang 2013). Yuting Hao et al. successfully purified DCP using a DEAE‐Cellulose‐52 column followed by a Sephadex G‐100 column (Hao et al. 2023). This approach is advantageous for its operational simplicity and avoidance of organic solvents, though scalability is limited.

Other methods include precipitation and membrane separation. Precipitation is straightforward and effective for polysaccharides with markedly different solubilities. Zhen‐Zi Shang et al. isolated three distinct polysaccharide fractions from *D. chrysotoxum* stems using stepwise ethanol precipitation at concentrations of 40%, 60%, and 80% (Shang et al. 2021). Membrane separation technology, which exploits molecular weight differences under pressure‐driven filtration, offers reusability, mild operating conditions, and preservation of bioactivity. Nevertheless, its application in DCP purification is still relatively uncommon.

## 3 Structural Characteristics of *D. chrysotoxum* Polysaccharides

Polysaccharides from *D. chrysotoxum* are heteropolysaccharides with diverse and complex structures. Their intricate chemical architecture underlies their wide range of biological functions. To better understand the pharmacological potential of DCP, it is necessary to analyze their structural features. Reported homogeneous fractions have been characterized in terms of monosaccharide composition, molecular weight, and chemical structure (Table 3).

**TABLE 3:** Structure of DCP.

Name	Monosaccharide compositions	Molecular weights (Mw, kDa)	Proposed structure	References
DCP‐H	Glc:Man:Ara:Gal:Xyl = 57.5:38.5:1.7:1.5:0.8	2240	NA	Pan et al. (2015)
DCP‐E	Glc:Man:Ara:Gal = 85.1:12.7:0.8:1.4	2010	NA	
DCP‐1	Glc:Man:Gal = 55.6:37.8:2.9	15	A 2/3‐O‐acetylglucomannan mainly consisted of (1→4)‐linked β‐ᴅ‐Manp and (1→4)‐linked β‐ᴅ‐Glcp (in 5:4 ratio)	Hao et al. (2023)
DCP‐W4	Glc:Man = 3.0:1.0	13.7	A glucomannan containing acetyl groups	Shang et al. (2021)
DCP‐W1	Glc:Man = 6.9:2.9	109.2	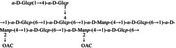	Wang (2013)
DCP‐W2	Glc:Man:Gal:Ara = 9.0:6.1: 1.0:0.9	1830	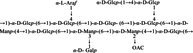	
DCP‐W3	Glc:Man:Gal:Xyl:Rha = 24.6:5.1:36.8:5.1:3.4	1960	A backbone consisted of 1,4‐linked GalAp and 1,2‐linked Rhap resides in the molar ratio of 1: 1 with occasional branches at O‐ 4 and O‐3	
DCP‐F4	Glc:Man:Xyl = 16:7:1	13.7	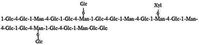	Xu (2017)

*Note:* NA: There are currently no literature reports available. Name: The names of polysaccharide fractions listed in the table are the original designations used in the respective references.

### 3.1 Monosaccharide Composition

Analysis of monosaccharide composition is the basis of polysaccharide structural research. Common methods include polysaccharide hydrolysis, derivatization, followed by quantitative detection using high‐performance liquid chromatography (HPLC) or gas chromatography (GC) (Liu et al. 2021). Current evidence suggests that polysaccharides isolated from *D. chrysotoxum* are primarily composed of glucose and mannose, with smaller amounts of galactose, arabinose, and xylose. The differences lie mainly in the molar ratios of these monosaccharides, which appear to be influenced by extraction and purification techniques. For example, ethanol extraction yielded a homogeneous fraction DCP‐W4 with a composition of Glc:Man = 3.0:1.0 (Sun et al. 2013). In another study comparing hot‐water extraction and enzyme‐assisted extraction, the hot‐water fraction DCP‐H had a composition of Glc:Man:Ara:Xyl:Gal = 57.5:38.5:1.7:0.8:1.5, whereas the enzyme‐assisted fraction DCP‐E consisted of Glc:Man:Ara:Gal = 85.1:12.7:0.8:1.41 (Pan et al. 2015). Notably, glucose content is consistently higher than mannose content, and variations in monosaccharide profiles are likely attributable to extraction and preparation methods.

### 3.2 Molecular Weight

Molecular weight is an important physicochemical characteristic of polysaccharides and a critical determinant of their biological activities. It is commonly measured by HPLC or high‐performance gel permeation chromatography (HPGPC). Reported molecular weights of DCP typically range from 10^4^ to 10^6^ Da (Hao et al. 2023; Pan et al. 2015; Shang et al. 2021; Sun et al. 2013). Substantial variations may arise from differences in raw material origin, extraction and purification techniques, and analytical methods. For instance, the molecular weight of DCP‐H obtained by hot‐water extraction (2240 kDa) was greater than that of DCP‐E extracted using enzymatic methods (2010 kDa) (Pan et al. 2015). The lower molecular weights observed in enzymatic and ultrasonic fractions are likely due to enzymatic hydrolysis of the plant cell wall or ultrasonic cavitation and mechanical disruption, which degrade polysaccharide chains (Pan et al. 2015; Sun et al. 2013).

### 3.3 Chemical Structure

In‐depth analysis of chemical structure provides molecular insights into the mechanisms underlying polysaccharide bioactivities. Studies have shown that DCP mainly consist of (1→4)‐linked Manp and (1→4)‐linked Glcp, with partial acetylation (Hao et al. 2023; Wang 2013; Xu 2017). Variations in plant parts used and extraction methods may lead to differences in linkage patterns and branching structures (Liu et al. 2020; Zhu et al. 2023). For example, Hao et al. reported that DCP‐1 is composed of a backbone of 1,4‐β‐D‐Manp and 1,4‐β‐D‐Glcp, with minor 1,4,6‐linked glucose residues as side chains (Hao et al. 2023). Xu et al. found that DCP‐F4 also contained a backbone of 1,4‐linked mannose and glucose, but with 1,4,6‐linked mannose residues as branches (Xu 2017). Wang et al. reported that DCP‐W1 and DCP‐W2 contained 1,6‐glycosidic linkages in their backbones, whereas DCP‐W3 displayed a distinct structure with a backbone consisting of 1,4‐linked galacturonic acid and 1,2‐linked rhamnose, accompanied by unique side‐chain linkages (Wang 2013). These comparisons illustrate that different extraction and purification methods applied by various researchers result in structural diversity among the obtained polysaccharide fractions.

Common techniques employed for polysaccharide structural elucidation include viscosity measurements, Fourier‐transform infrared (FT‐IR) spectroscopy, methylation analysis coupled with gas chromatography–mass spectrometry (GC–MS), and nuclear magnetic resonance (NMR) spectroscopy (Hao et al. 2023; Pan et al. 2014; Sun et al. 2013). In addition, Jiang et al. established a method using direct analysis in real‐time mass spectrometry (DART‐MS) for real‐time characterization of herbal polysaccharides. This approach enables rapid fragmentation of polysaccharides into ions with m/z < 350, producing characteristic spectra that can be used to effectively differentiate polysaccharides from different herbal sources (Jiang and Li 2021). Specifically, this method has been successfully applied to the detection of DCP, with the estimated molecular weight of DCP being 1.82 × 10^3^ kDa and its major characteristic fragment ions identified as m/z 127.04, 145.05, 187.06, and 205.07 in the same study (Jiang and Li 2021).

## 4 Biological Activities and Mechanisms of *D. chrysotoxum* Polysaccharides

**FIGURE 2:**
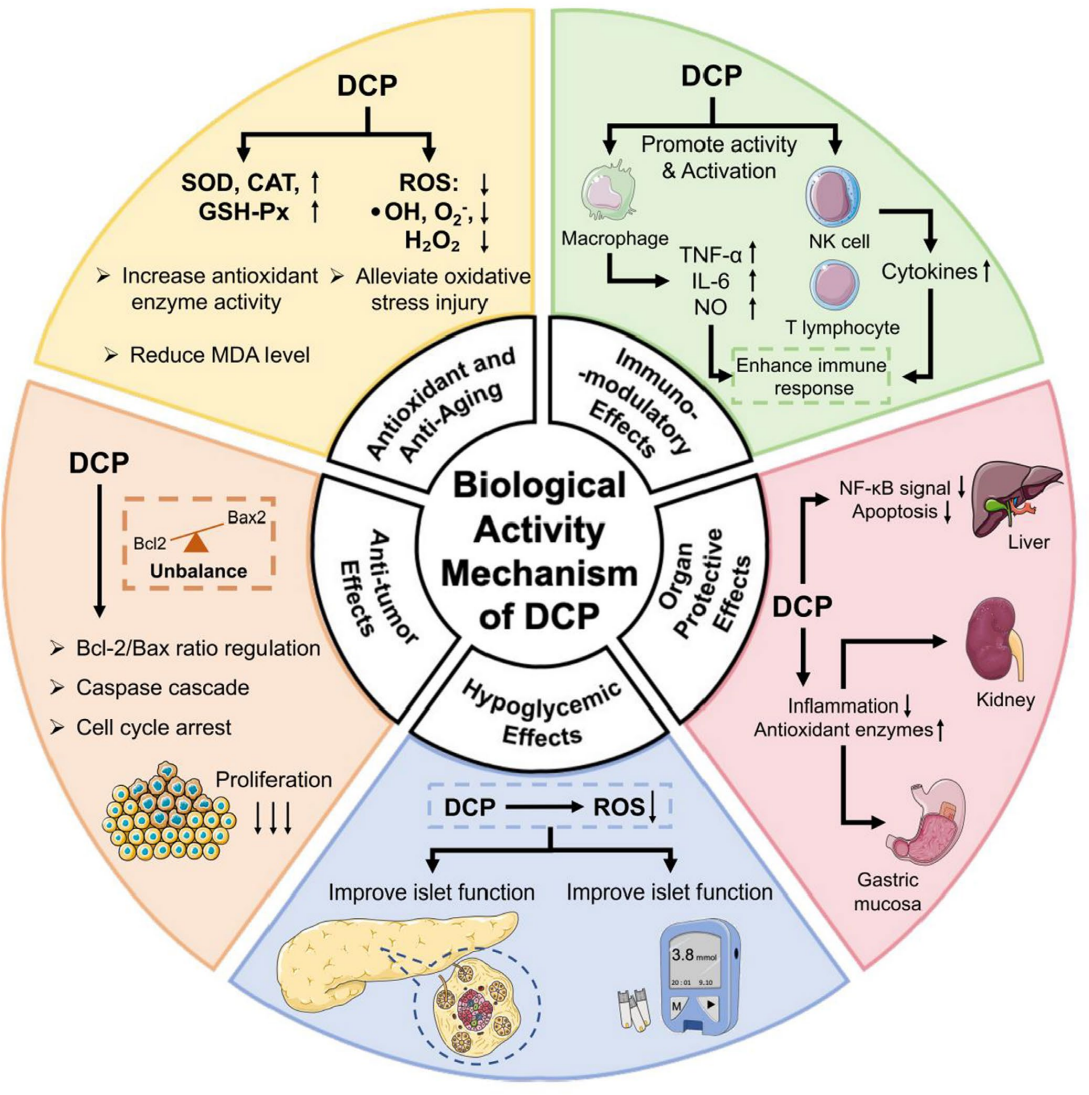
The biological activity mechanism of DCP.

Recent advances in polysaccharide research have revealed that *Dendrobium* species are rich sources of biologically active polysaccharides with diverse pharmacological properties, including antioxidant, immunomodulatory, hypoglycemic, and antitumor activities (Figure 2). Among them, polysaccharides isolated from *Dendrobium officinale* (DOP) have been extensively investigated and reported to exert multiple biological functions such as immune regulation, antioxidant activity, and antitumor effects through various molecular mechanisms (Bian et al. 2025; He et al. 2016; Pang et al. 2024). These studies highlight the significant therapeutic potential of *Dendrobium*‐derived polysaccharides as a whole. In comparison, research focusing specifically on polysaccharides from *D. chrysotoxum* (DCP) remains relatively limited and has only recently begun to receive increasing attention. In this chapter, we systematically review the antioxidant and anti‐aging, immunomodulatory, organ‐protective, hypoglycemic, and antitumor activities of DCP, emphasizing both their shared mechanisms with other *Dendrobium* polysaccharides and their unique biological characteristics.

### 4.1 Antioxidant and Anti‐Aging Effects

Oxidative stress is a major contributor to aging, chronic inflammation, immune dysfunction, and the development of diseases such as diabetes and cancer. The antioxidant research of *Dendrobium* medicinal plants has been more extensive. *D. officinale* , as one of the representative plants, its main component DOP can play a significant antioxidant role, so as to alleviate atherosclerosis and protect nerves (Liang et al. 2019; Qi et al. 2024). As a commonly used medicinal herb, multiple studies have reported the antioxidant and anti‐aging effects of polysaccharides derived from *D. chrysotoxum*. These polysaccharides exhibit strong free‐radical scavenging capacity and overall antioxidant activity, including the ability to scavenge hydroxyl radicals, superoxide anions, and DPPH radicals in a dose‐dependent manner (Zhao et al. 2007; Xie et al. 2022; Xu et al. 2025).

Comparative studies have demonstrated that DCP possess effective antioxidant properties. Chen et al. (2022) found that among eight *Dendrobium* species, *D. chrysotoxum* showed superior activity in certain aspects. In vitro antioxidant activity assays demonstrated that the DPPH radical scavenging rate of DCP reached 23.87%, which was significantly higher than that of the other six *Dendrobium* species. Meanwhile, when determined by the ABTS assay, the total antioxidant capacity of DCP exhibited a high scavenging efficiency of 81.40%, and its total antioxidant activity was also distinctly superior to that of polysaccharides from other tested *Dendrobium* species (Chen et al. 2022). These findings suggest that DCP can effectively attenuate oxidative stress by scavenging free radicals and suppressing lipid peroxidation. Consistent with the antioxidant mechanisms illustrated in Figure 2, DCP has been reported to enhance endogenous antioxidant defenses by increasing superoxide dismutase (SOD) activity and reducing intracellular reactive oxygen species (ROS) levels. Mechanistically, as an O‐acetylated glucomannan, DCP is suggested to promote the nuclear translocation of Nrf2, thereby inducing the transcription of antioxidant‐related genes, including SOD, and ultimately upregulating the expression and activity of endogenous antioxidant enzymes (Hao et al. 2023).

Oxidative stress is a major driver of aging, and the antioxidant capacity of DCP can alleviate oxidative damage to organisms, thereby exerting anti‐aging effects. Interestingly, DCP were also shown to markedly extend the lifespan of *Caenorhabditis elegans* without compromising reproductive capacity. In addition, they improved locomotive behavior and acute heat stress resistance in nematodes in a dose‐dependent manner (Li et al. 2022).

### 4.2 Immunomodulatory Effects

The immune system is a crucial physiological barrier for maintaining homeostasis and defending against pathogenic invasion. Studies have reported that DOP can play an immunomodulatory role through TLR4‐NF‐кB and other multiple pathways, and has a wide range of immunomodulatory abilities, such as promoting the release of secretory IgA (sIgA) and regulating the secretion of cytokines (Guo et al. 2022; Huang, He, et al. 2018; Li et al. 2024; Nie et al. 2025; Xie et al. 2022; Zhou et al. 2025). Remarkably, studies have shown that DCP also exhibit strong intestinal immunomodulatory activity in vitro. Crude extracts enhanced bone marrow cell proliferation by 3.3%–89.0% (Xu 2017). Through stepwise fractionation, a highly active fraction (DCP‐F4) was obtained, which stimulated bone marrow cell proliferation at 2.136 times that of the control (Shang et al. 2021). Further studies revealed that DCP‐F4 significantly influenced immune activity by altering the indices of immune organs (spleen and thymus), promoting bone marrow cell proliferation mediated by Peyer's patch supernatants, balancing the Th1/Th2 ratio through regulation of IFN‐γ and IL‐4 secretion, modulating the proportion and differentiation of immune cells (T cells, dendritic cells, and macrophages), and increasing the production of sIgA, β‐defensin, and mucin‐2 in the intestine and spleen. Moreover, DCP were found to promote splenocyte proliferation in mice in a dose‐dependent manner (Pan et al. 2015). Importantly, they enhanced macrophage functions through multiple mechanisms, including phagocytosis, nitric oxide (NO) release, and secretion of cytokines such as IL‐1α, IL‐6, IL‐10, and TNF‐α (Meng et al. 2013).

DCP exhibit context‐dependent immunomodulatory effects, capable of both stimulating immune responses under basal conditions and suppressing excessive inflammation in activated states. Another study demonstrated that aqueous extracts of *D. chrysotoxum* significantly inhibited NO production in a concentration‐dependent manner. They also reduced the secretion of TNF‐α, monocyte chemoattractant protein‐1 (MCP‐1), and IL‐6. Further analysis revealed that the extracts suppressed the expression of iNOS and COX‐2, as well as the phosphorylation of ERK and JNK, suggesting that the inhibitory effect on LPS‐induced macrophage inflammation was mediated through suppression of the MAPK pathway. These findings are consistent with the immunomodulatory mechanisms summarized in Figure 2, where DCP regulate macrophage activation and cytokine production through multiple signaling pathways (Zeng et al. 2018).

### 4.3 Organ Protective Effects

Traditionally, *D. chrysotoxum* has been used to “nourish the stomach and promote fluid production” and to “moisten the lung and replenish Yin” (Lee et al. 2018). Modern studies have preliminarily confirmed its organ‐protective effects. Current evidence indicates that DCP and other *Dendrobium* polysaccharides exert protective effects on multiple organs, including the kidney, liver, and gastric mucosa, primarily through the regulation of oxidative stress and the suppression of inflammatory responses (Hao et al. 2023; Zhang et al. 2024).

Polysaccharides from *D. chrysotoxum* have demonstrated protective effects in renal injury models. In a mouse model of LPS‐induced acute kidney injury (AKI), administration of DCP alleviated both inflammation and tubular damage. Biochemical analyses showed significant reductions in malondialdehyde (MDA), tumor necrosis factor‐α (TNF‐α), interleukin‐6 (IL‐6), and cyclooxygenase‐2 (COX‐2) levels, indicating a strong suppression of oxidative stress and inflammatory responses. Histological examination further confirmed attenuation of tubular epithelial cell swelling, necrosis, and infiltration of inflammatory cells. These findings suggest that DCP protects renal function not only by reducing oxidative stress but also by modulating inflammatory signaling pathways, thereby preventing tissue injury and promoting structural integrity of renal tissue (Hao et al. 2023).

The hepatoprotective activity of DCP has been verified in several models of liver injury. Experimental data indicate that DCP mitigates tissue injury by attenuating oxidative stress and inflammatory responses, as evidenced by reduced MDA levels, enhanced SOD activity, and downregulation of pro‐inflammatory cytokines (e.g., TNF‐α and IL‐6), suggesting potential hepatoprotective effects (Hao et al. 2023). Moreover, DCP were shown to modulate lipid metabolism, leading to reductions in serum triglyceride and cholesterol levels, and to inhibit the accumulation of lipid droplets in hepatocytes. In addition to their antioxidative effects, they exert pronounced anti‐inflammatory actions by suppressing NF‐κB activation, downregulating pro‐inflammatory mediators such as TNF‐α and IL‐6, and decreasing expression of inducible nitric oxide synthase (iNOS). Importantly, DCP inhibit hepatocyte apoptosis via regulation of Fas/FasL signaling and by modulating the Bcl‐2/Bax ratio, thereby preserving cell viability and liver function. Collectively, these findings suggest that the hepatoprotective mechanism of DCP involves a combination of antioxidant, anti‐inflammatory, lipid‐regulating, and anti‐apoptotic activities, highlighting their potential as natural agents for liver protection (Qian et al. 2015).

DCP have also been reported to exert protective effects on gastric mucosa, primarily through antioxidant and anti‐inflammatory mechanisms. In vitro studies using GES‐1 gastric epithelial cells demonstrated that treatment with polysaccharides markedly increased the activities of endogenous antioxidant enzymes, including SOD, CAT, and GSH‐Px, while significantly reducing intracellular ROS levels. These changes contributed to the stabilization of redox balance and enhanced cellular resistance against oxidative damage. Furthermore, polysaccharide administration was found to decrease lipid peroxidation products such as malondialdehyde (MDA), thereby preventing membrane damage and maintaining epithelial integrity (Wang et al. 2024).

In vivo observations provided further evidence of gastric protective effects. Histopathological evaluation showed that DCP attenuated mucosal edema, epithelial shedding, and inflammatory cell infiltration in gastric tissues subjected to chemical or oxidative injury. In addition, their regulatory role on cytokine secretion suggested that the protective effect may also be mediated by suppression of local inflammatory responses (Zhao et al. 2007). Interestingly, protective actions were not limited to the gastric mucosa, as some studies reported concurrent improvements in pancreatic tissue morphology, implying a broader role in gastrointestinal protection (Pan et al. 2014). Collectively, these findings highlight that DCP protects the gastric mucosa through a synergistic mechanism involving enhancement of antioxidant defenses, suppression of oxidative stress–induced injury, and regulation of inflammatory signaling (Wang et al. 2024).

The results of the above research coincide with the existing research reports that DOP has digestive tract protection and heart protection, indicating that different species of *Dendrobium* have similar organ protection functions, which has great application potential (Han et al. 2025; Zhang et al. 2019; Zhu et al. 2024).

### 4.4 Hypoglycemic Effects

Hyperglycemia is recognized as the central pathogenic factor driving both the onset and progression of diabetes and its related complications (Yu et al. 2021). While studies on DOP have demonstrated its efficacy in mitigating diabetes progression, alleviating complications, and stimulating GLP‐1 secretion, recent investigations into DCP have also yielded exciting and encouraging findings regarding its hypoglycemic activity (Chen et al. 2023; Kuang et al. 2020; Zhang et al. 2024). Experimental studies have demonstrated that *Dendrobium chrysotoxum* Lindl. polysaccharide (DCP), when administered at doses ranging from 200 to 500 mg/kg, significantly lowered blood glucose levels in alloxan‐induced diabetic mice. Beyond their direct hypoglycemic action, these polysaccharides also exhibited potent free radical scavenging activity against hydroxyl radicals and superoxide anions, thereby alleviating oxidative stress—a major contributor to β‐cell dysfunction and insulin resistance (Zhao et al. 2007).

Although the hypoglycemic effect of DCP was somewhat weaker than that of *D. officinale* and *D. huoshanense*, higher doses of DCP were associated with marked reductions in malondialdehyde (MDA) levels, suggesting an ability to inhibit lipid peroxidation and prevent oxidative damage in pancreatic tissue (Pan et al. 2014). Taken together, these findings indicate that the hypoglycemic activity of DCP is not only due to improved glucose regulation but also involves enhancement of antioxidant defense mechanisms, thereby providing a dual protective effect against diabetes‐induced metabolic and oxidative injury.

### 4.5 Antitumor Effects

Polysaccharides extracted from *D. chrysotoxum* have demonstrated significant inhibitory effects on the proliferation of SPC‐A‐1 lung cancer cells and HeLa cervical cancer cells in vitro, and these effects were clearly dose‐dependent (Li 2008). Comparative analyses further revealed that refined polysaccharide fractions exerted stronger antiproliferative activity than crude extracts, highlighting the importance of purification and structural integrity in determining biological efficacy (Sun et al. 2013).

In comparison, polysaccharides from *Dendrobium* officinale (DOP) have been more extensively studied and are known to suppress tumor cell proliferation and metastasis through multiple mechanisms. For example, DOP‐based carriers can significantly enhance the efficacy of photodynamic therapy (Tao et al. 2023). Moreover, DOP can play an anti‐tumor role by inducing cell apoptosis and promoting the activity of macrophages and NK cells to enhance immune‐mediated tumor inhibition (Ruan et al. 2023; Tao et al. 2025; Wang et al. 2022; Zhang et al. 2021). Although studies on DCP remain limited, its structural similarity and shared biological properties with DOP suggest that DCP may exert comparable antitumor activities. Excitingly, this area remains largely unexplored, offering substantial potential for future mechanistic and translational research.

Mechanistic investigations suggest that the antitumor activity of DCP may be closely related to their structural features—particularly monosaccharide composition, glycosidic linkages, and molecular weight distribution. At the cellular level, these polysaccharides are thought to trigger apoptosis by activating the caspase cascade, modulating the Bcl‐2/Bax ratio, and disrupting mitochondrial membrane potential. In addition, they may influence cell cycle progression by regulating cyclin‐dependent kinases (CDKs), ultimately inducing cell cycle arrest (Wei et al. 2018). Collectively, these findings indicate that DCP might possess multi‐target antitumor potential through a combination of apoptosis induction, oxidative stress modulation, and cell cycle regulation.

## 5 Conclusions and Future Perspective

Research on DCP has established a strong foundation for their future development, encompassing advances in extraction, purification, structural characterization, and biological evaluation. Extraction methods have progressed from traditional hot‐water extraction with ethanol precipitation to modern assisted techniques such as enzyme‐, ultrasonic‐, and microwave‐assisted extraction. Factors including temperature, liquid‐to‐solid ratio, and raw material pretreatment markedly affect extraction efficiency. Purification typically involves deproteinization, dialysis, and chromatographic fractionation (e.g., DEAE‐cellulose, Sephadex G‐series) to obtain homogeneous fractions. Structurally, DCP are heteropolysaccharides mainly composed of glucose and mannose, with molecular weights of 10^4^–10^6^ Da, (1→4)‐linked Manp and Glcp backbones, branching, and partial acetylation. Biologically, DCP exhibit antioxidant, immunomodulatory, organ‐protective, hypoglycemic, and antitumor activities, showing promise for pharmaceutical and functional food applications.

Among these activities, oxidative stress regulation appears to be a central upstream mechanism linking multiple biological effects. In metabolic disorders such as diabetes, oxidative stress contributes to pancreatic β‐cell dysfunction, insulin resistance, and impaired glucose metabolism. Previous studies on *Dendrobium* polysaccharides have demonstrated that the reduction of reactive oxygen species and inflammatory mediators can indirectly improve glucose homeostasis and pancreatic function. Therefore, in the schematic diagram (Figure 2), antioxidant mechanisms are summarized as an independent module, while the hypoglycemic section focuses on the direct physiological outcomes related to glucose regulation to avoid redundancy in mechanistic illustration.

However, several challenges persist. Conventional extraction remains inefficient and energy‐intensive, while assisted methods face issues of cost, temperature control, and scalability. Decolorization, a key yet often overlooked step, can cause polysaccharide loss or structural alteration. Most biological studies remain descriptive, lacking mechanistic depth and clear structure–activity relationships (SAR). Moreover, the absence of standardized quality control indices (e.g., acetylation degree, activity markers) limits reproducibility and industrial application. If DCP were to be commercialized, they would fall into different global regulatory categories depending on the intended use and region, such as dietary supplements (e.g., USA), novel foods (e.g., EU), or botanical drugs (e.g., China). Key challenges for regulatory approval include the lack of standardized quality control protocols, batch‐to‐batch consistency, comprehensive safety and toxicity evaluation, and elucidation of active marker compounds.

Future efforts should prioritize three directions. (1) Development of green and efficient extraction methods such as freeze–thaw, subcritical water, and deep eutectic solvent (DES)‐based extraction to enhance yield, preserve bioactivity, and reduce environmental impact. (2) Elucidation of molecular mechanisms and SAR through integrated biochemical, omics, and modeling approaches to clarify key functional motifs linked to activity. (3) Industrial translation via the creation of targeted functional products (e.g., hepatoprotective, anti‐aging, hypoglycemic formulations) and establishment of standardized quality systems. The integration of enzymatic hydrolysis with membrane separation can further improve efficiency and batch consistency.

Overall, advancing green extraction technologies, mechanistic understanding, and standardization will accelerate the transition of DCP from basic research to high‐value applications, realizing their potential as multifunctional natural biomacromolecules.

## Author Contributions

**Weitong Zhang:** validation. **Zhaoyuan Xu:** methodology, investigation, data curation. **Xingshuai Zhang:** methodology, data curation, investigation, writing – review and editing, writing – original draft. **Jialin Chen:** resources, formal analysis. **Lihang Xie:** conceptualization, writing – review and editing, funding acquisition, resources, project administration, methodology, supervision.

## Funding

This work was supported by the National Natural Science Foundation of China (Grant No. 32200215) and the postdoctoral research grant in Henan Province (Grant No. 202103024).

## Conflicts of Interest

The authors declare no conflicts of interest.

## Data Availability

No data availability statement is required separately for the manuscript. All the data have been made available in the article.
